# Pressor threshold of muscle metaboreflex is modulated during unloading of carotid baroreceptors in humans

**DOI:** 10.1186/2046-7648-4-S1-A62

**Published:** 2015-09-14

**Authors:** Masashi Ichinose, Tomoko Ichinose, Kazuhito Watanabe, Takeshi Nishiyasu

**Affiliations:** 1 Human Integrative Physiology Laboratory, School of Business Administration, Meiji University, Tokyo, Japan; 2 Laboratory for Human Performance Research, Osaka International University, Osaka, Japan; 3 Institute of Health and Sports Science, University of Tsukuba, Tsukuba, Japan

## Introduction

Static and dynamic exercise are accompanied by increases in arterial blood pressure, heart rate and sympathetic nerve activity. It is thought that the activation of the muscle metaboreflex is one of major mechanisms for evoking the pressor responses during heavy intensity exercise [1]. It has been shown that the arterial baroreflex buffers the muscle metaboreflex-mediated pressor responses [2]. However, it is unknown whether the carotid baroreflex modifies muscle metaboreflex function in humans. Therefore, the purpose of this study was to investigate the effects of unloading of carotid baroreceptors on threshold and gain of the muscle metaboreflex in humans.

## Methods

Subjects (nine males and one female with a mean age of 23 (2) years, a body weight of 63.2 (3.0) kg, and a height of 171.0 (2.8) cm.) performed static handgrip exercise at 50% of maximum voluntary contraction. The contraction was sustained for 15, 30, 45 and 60 s, followed by 3 min of circulatory arrest, respectively. It has been demonstrated that in this maneuver, forearm muscular pH during the ischemia linearly decreases with increasing contraction time [3]. The carotid baroreceptors were unloaded by 0.1 Hz sinusoidal neck pressure (oscillates from 15 to 50 mmHg) during the third min of ischemia. We compared cardiovascular responses during the ischemia with and without unloading of the carotid baroreceptors. In addition, we estimated the threshold and gain of the muscle metaboreflex by analyzing the relationship between cardiovascular responses during the third min of ischemia and amount of work during handgrip (i.e., integrated values of handgrip force).

## Results

During unloading of carotid baroreceptors, the muscle metaboreflex thresholds for mean arterial blood pressure (MAP) and for total vascular resistance (TVR) located significantly lower work amount compared to those in control conditions (threshold for MAP: 795 (32) vs. 662 (66), for TVR: 818 (67) vs. 572 (92) kg.s, p < 0.05). The gains of the muscle metaboreflex that were estimated as maximum rate of changes in hemodynamic values to change in work amount were not different between these two conditions (gain for MAP: 4.9 (0.5) vs. 4.4 (0.5) mmHg.kg.s^-1^.100, for TVR: 1.3 (0.3) vs. 1.3 (0.2) mmHg.L^-1^.min^-1^.kg.s^-1^.100).

## Discussion

Our results show that unloading of carotid baroreceptors lowers the pressor threshold of the muscle metaboreflex in humans. This indicates that under normal blood pressure conditions, the carotid baroreflex shifts the threshold to a higher metabolic stimulation level and thus inhibits the muscle metaboreflex mediated pressor response.

## Conclusion

We conclude that the carotid baroreflex modifies the muscle metaboreflex threshold in humans. We suggest that the modulation of the muscle metaboreflex function through carotid baroreflex would contribute to cardiovascular regulations during exercise.
